# Silver nanoparticles improve the fungicidal properties of *Rhazya stricta* decne aqueous extract against plant pathogens

**DOI:** 10.1038/s41598-024-51855-5

**Published:** 2024-01-14

**Authors:** Sarah A. Al-Sahli, Fatimah Al-Otibi, Raedah I. Alharbi, Musarat Amina, Nawal M. Al Musayeib

**Affiliations:** 1 Department of Botany and Microbiology, College of Science, King Saud University, P.O. Box 22452, 11495 Riyadh, Saudi Arabia; 2 Department of Pharmacognosy, Pharmacy College, King Saud University, 11451 Riyadh, Saudi Arabia

**Keywords:** Experimental organisms, Developmental biology, Microbiology, Bacteriology, Environmental microbiology, Fungi

## Abstract

One of the most promising, non-toxic, and biocompatible developments for many biological activities is the green synthesis of nanoparticles from plants. In this work, we investigated the antifungal activity of silver nanoparticles (AgNPs) biosynthesized from *Rhazya stricta* aqueous extract against several plant pathogenic fungi. UV–visible spectroscopy, Zeta potential analysis, Fourier-transform infrared spectroscopy (FTIR), and transmitted electron microscopy (TEM) were used to analyze the biosynthesized AgNPs. *Drechslera halodes, Drechslera tetramera, Macrophomina phaseolina, Alternaria alternata,* and *Curvularia australiensis* were tested for their potential antifungal activity. Surface Plasmon Resonance (SPR) of Aq. AgNPs and Alkaline Aq. AgNPs was observed at 405 nm and 415 nm, respectively. FTIR analysis indicated hydroxyl, nitrile, amine, and ketone functional groups. Aq. AgNPs and Alka-line Aq. AgNPs had velocities of − 27.7 mV and − 37.9 mV and sizes of 21–90 nm and 7.2–25.3 nm, respectively, according to zeta potential studies and TEM. The antifungal examination revealed that all species' mycelial development was significantly inhibited, accompanied by severe ultra-structural alterations. Among all treatments, Aq. AgNPs were the most effective fungicide. *M. phaseolina* was statistically the most resistant, whereas *A. alternata* was the most vulnerable. To the best of our knowledge, this is the first report on *R. stricta'*s antifungal activity against these species.

## Introduction

Medicinal plants are high in phytochemicals and antibacterial compounds. Various medicinal plant components are harvested for their diverse therapeutic properties^1^. Medicinal plants have been utilized in folk medicine in Saudi Arabia and the rest of the Arabian Peninsula throughout the dawn of time to cure a variety of ailments^2^. This region has a collection of wild medicinal plants, making it a natural reservoir, and accounts for around 27% of the flora that has been documented to be medicinally valuable^3,4^.

*Rhazya stricta* Decne is a perennial evergreen dwarf poisonous shrub with a smooth central stem and thick semi-erect branches that belongs to the *Apocynaceae* family and is extensively found in the Middle East and Indian subcontinent^5^. *R. stricta* thrives in depressions with silty and sandy soils, establishing a pure stand at times. It also grows in rocky terrain, hills, plains, and wadis. *R. stricta* is found in the sandy plains of Saudi Arabia^5,6^. Plants in this family are widely recognized for their unique array of terpenoid indole alkaloids with different biological activity, as well as flavonoids, glycosides, triterpenes, and tannins^6^. *R. stricta* has been used to treat a variety of ailments, including fever and chronic rheumatism^7^. Indole alkaloids have several biological actions, including anticancer, antibacterial, and antihypertensive effects, as well as being central nervous system stimulants^8^. The existence of 20 monomeric terpenoid indole alkaloids with molecular weights ranging from 278 to 354 was discovered in *R. stricta* hairy root extracts. These included aspidospermine, eburnamine, aspidospermatin, pleiocarpaman, strychnos, sarpagine, heteroyohimbine, yohimbinoid, and hunterburine alkaloids. According to GC–MS analysis, eburenine is the most abundant alkaloid in the hairy roots and leaves of *R. stricta*^9^.

Maladies of plants that spread through seeds are known as seed-borne diseases. One of the major culprits is seed-borne fungus. They may reduce seed germination, weaken germs, negatively impact freshly growing plants, and taint the soil^10^. Wheat seed-borne diseases are caused by harmful types of fungi, such as *Alternaria alternata, Cladosporium herbarum, Drechslera sorokiniana*, and *Drechslera tetramera*, which can cause yield losses if not treated^11^. Other phytopathogenic fungi transmitted through soil (soil-borne fungus), such as *Macrophomina phaseolina*, have been shown to infect over 500 plant species. These pathogens cause various diseases, including stem and root rot, charcoal rot, and seedling blight^12^.

The unregulated and random use of pesticides in agriculture caused several environmental issues, including pollution of water, soil, animals, and food, as well as the unintended extermination of non-target species and phytopathogens^13^. As a result, alternative methods for controlling plant disease and minimizing the harmful effects of synthetic fungicides have been developed, including biological control through the use of natural compounds having antimicrobial properties. This includes the use of medicinal plant extracts and essential oils^14^.

Because of their unique optical, magnetic, electrical, and chemical capabilities, nanoparticles have been employed in a wide range of applications, including solar cells, photovoltaic devices, heterogeneous catalysts, and medicine^15^. Nanoparticles have the potential to saturate and stick to the surface of fungal hyphae, preventing harmful fungi from growing^16^. Because of the stabilizing factors that easily allow nanoparticles to connect with other biomolecules and increase their interactions with bacteria, biologically generated nanoparticles have higher antimicrobial efficacy^17^. Biogenic silver nanoparticles (AgNPs), for example, had more antibacterial activity than chemically generated nanoparticles^18^. By interacting with proteins and enzymes, AgNPs can induce persistent cell damage by disrupting the electron transport chain, resulting in membrane permeability barrier disruption^19^. Examples for the biogenic activities of biosynthesized AgNPs from different plants extracts highlighted their antimicrobial, anticancer, and apoptosis inducing ability. For example, a pervious study showed that AgNPs synthesized from the *Lagerstroemia speciosa* (L.) Pers. flower buds had antimicrobial activity against *Staphylococcus aureus*, *Escherichia coli*, *Candida albicans*, and *Candida glabrata*, besides, its significant anticancer activity against MG-63 cells of Osteosarcoma^20^. Another study used AgNPs bio-fabricated from the aqueous extract of *Ixora brachypoda* leaves and showed antifungal activity against *Bacillus subtilis, Pseudomonas aeruginosa, E. coli, S. aureus, C. albicans, Fusarium oxysporum,* and *A. alternata*^21^.

So, the current work attempted to evaluate the antifungal capabilities of AgNPs biosynthesized from *R. stricta* aqueous extract and its alkaline fraction against certain plant pathogenic fungi. *Drechslera halodes, D. tetramera, M. phaseolina, A. alternata,* and *Curvularia australiensis* were the species evaluated.

## Results

### AgNPs were successfully biosynthesized

The extraction of *R. stricta* leaves yielded a semi-solid dark green aqueous extract and its alkaline fraction (Dark brown) with yields of 40 g and 30.1g, respectively. Both preparations were light yellow when dissolved in distilled water for AgNPs synthesis. The biosynthesized AgNps displayed a dramatic serial color shift from bright yellow to yellow, brown, and eventually dark brown, indicating silver ion reduction and full production of stable silver ions. Various spectroscopy and microscopic examinations were used to characterize biosynthesized AgNPs.

**Figure 1:**
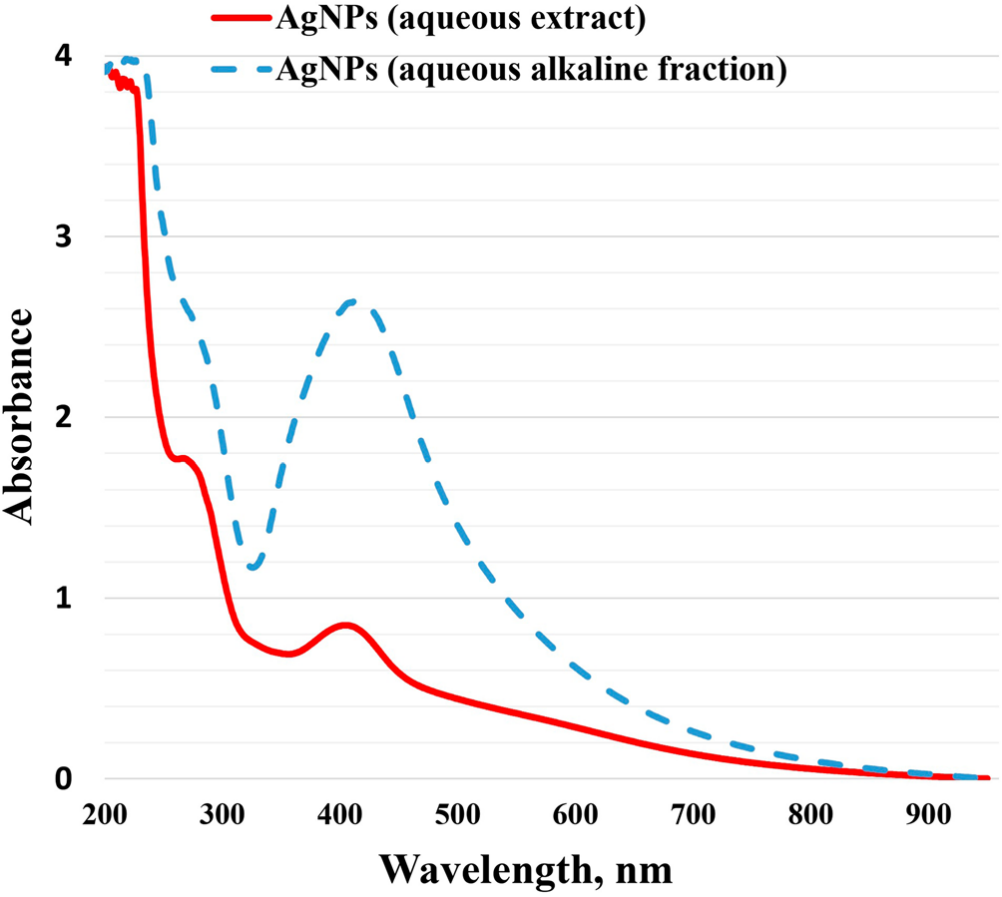
Results of the UV-vis spectra of AgNPs synthesized from *R. stricta* leaf extract. The UV-2600i spectrophotometer was used according to the manufacturer's recommendations for both the Aqueous AgNPs and Alkaline Aqueous AgNPs.

AgNPs' UV–visible spectra were measured between 200 and 900 nm. The UV–Vis spectra of Aq. AgNPs and Alkaline Aq. AgNPs resulted in two broad peaks at 405 and 415 nm, respectively (Fig. 1). As shown in Fig. 1, the peak of Alkaline Aq. AgNPs were higher than that of aqueous AgNPs, which might be attributed to increased energy consumption by the nanoparticles due to complicated bonding.

**Figure 2:**
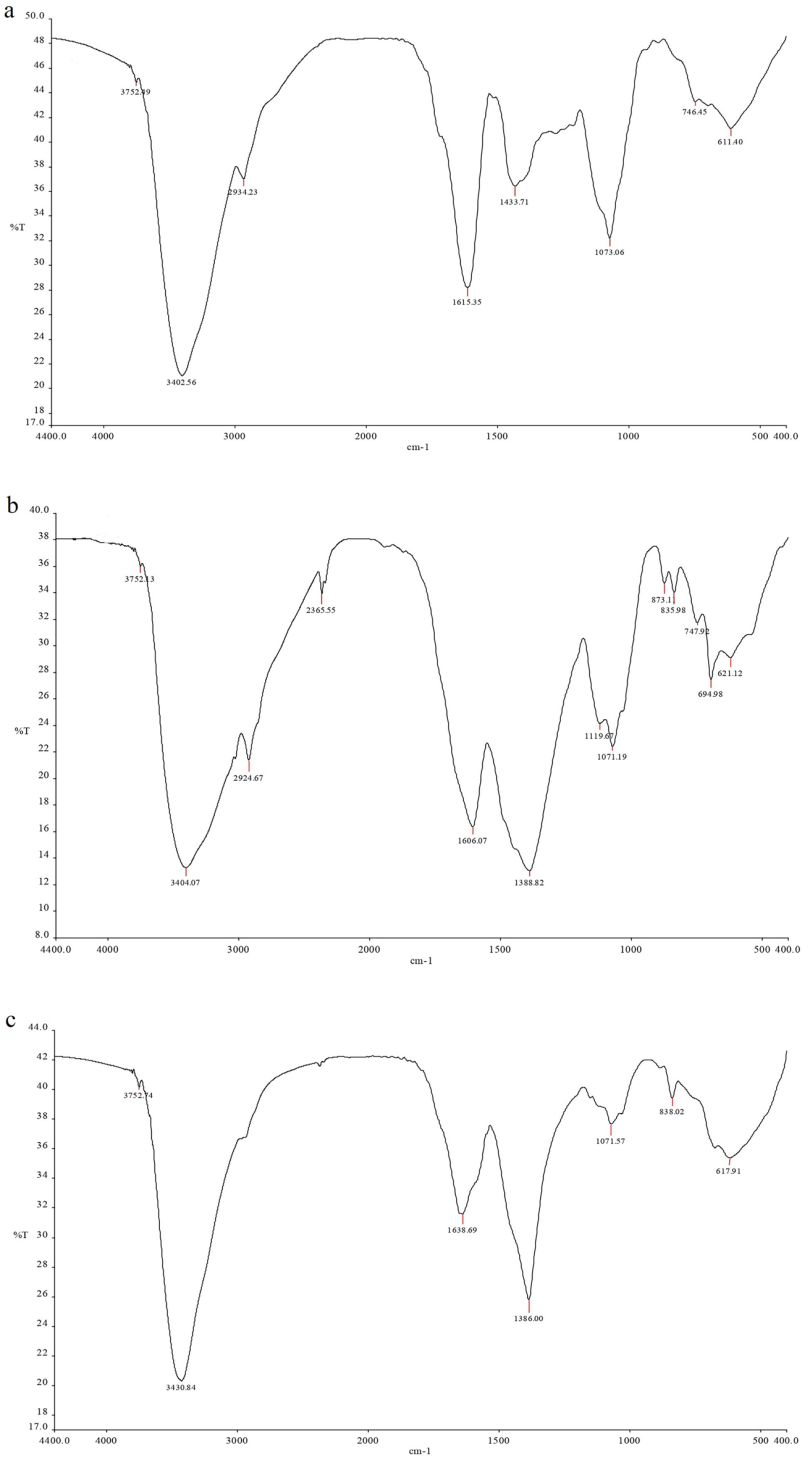
FTIR spectra of preparations of *R. stricta* leaves. Aqueous extract (**a**), Aqueous AgNPs (**b**), and Alkaline Aqueous AgNPs (**c**).

The FTIR spectra of *R. stricta* aqueous extract and biosynthesized AgNPs were analyzed to determine the nature of active ingredients responsible for nanoparticle reduction, stability, and bio-capping (Table 1 and Fig. 2). The presence of both bonded and non-bonded hydroxyl groups was indicated by broad peaks at 3402–3431 cm^−1^ and very weak peaks at 3402 cm^-1^ in the FTIR spectra of *R. stricta* preparations. Furthermore, all preparations showed peaks around 611–695 cm^−1^, indicating the existence of aliphatic bromo compounds. Methylene, aromatic rings, methyl, and cyclic ethers were found in both the crude extract and aq. AgNPs. The FTIR study of aqueous AgNPs indicated two distinct functional groups for nitrile (2365.55 cm^−1^) and secondary alcohol (1119.67 cm^−1^) compounds. At 836–897 cm^−1^, the aq. AgNPs and Alkaline Aq. AgNPs indicated the presence of peroxide. Table 1 shows that only the Alkaline Aq. AgNPs possessed four distinct functional groups for Quinone or conjugated ketone (at 1635.69 cm^−1^), sulfate (at 1386.00 cm^−1^), and amines (at 1242.06 and 1071.57 cm^−1^).

**Table 1:** FTIR analysis of *R. stricta* prepared materials in the current study.

Peak assignment	Functional group	Aq. extract	Aq. AgNPs	Alkaline Aq. AgNPs
Nonbonded hydroxyl	OH stretch	3752, vw	3752, vw	3752, vw
Hydroxy group	H-bonded OH stretch	3402, b	3404, b	3430, b
Methylene	C-H asym. /sym. stretch	2934, w	2924, w	–
Nitrile	CΞN stretch	–	2365, w	–
Conjugated ketone	C = O stretch	–	–	1635, m
Aromatic ring	C = C–C	1615, vs	1606, vs	–
Methyl	C-H asym. /sym. Bend	1433, m	1388, s	–
Sulfate	S = O stretch	–	–	1386, s
Amine	C-N stretch	–	–	1242; 1071, m
Secondary alcohol	C-O stretch	–	1119, vw	–
Cyclic ethers	C-O stretch large rings	1073, m	1071, m	–
Peroxides	C-O–O- stretch	–	873; 835, w	838.02, w
Methylene	–(CH2) n–	746, vw	747, w	–
Aliphatic bromo	C–Br stretch	611, w	694; 621, vw	617.91, b

*b* broad, *m* medium, *s* strong, *w* weak, *vs* very strong, *vw* very weak.

**Figure 3:**
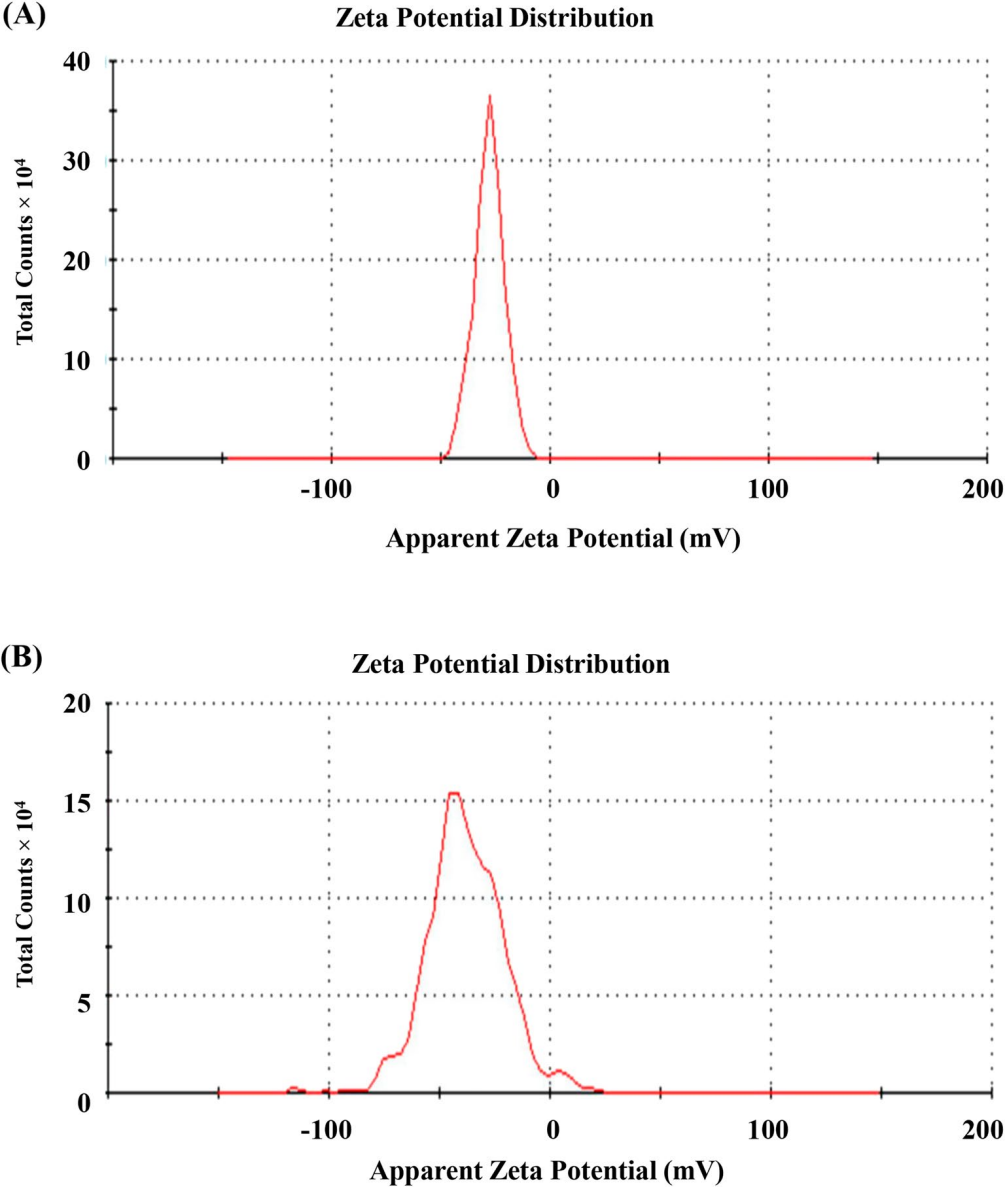
Zeta potential distribution of *R. stricta* leaves formed AgNPs. (**A**) Aqueous AgNPs; (**B**) Alkaline Aqueous AgNPs.

By calculating the velocity of the nano-sized particles, zeta potential analysis may be used to measure the surface charge and stability of the formulation of biosynthesized AgNPs. The velocity of nanoparticles is determined by their movement towards electrodes under the influence of an applied electric field. The Zeta potential of AgNPs produced by various *R. stricta* extracts and fractions was measured. The results showed that the A. AgNPs had a − 27.7 mV and the Alkaline Aq. AgNPs had a − 37.9 mV. These results demonstrated that the AgNPs produced are stable (Fig. 3). The average particle size, diameter, and polydispersity indices (PDI) of all pre-synthesized AgNPs, on the other hand, were evaluated. The average particle sizes (z-average) of Aq. AgNPs and Alkaline Aq. AgNPs were 95.9 nm (PDI value 0.220, intercept 0.874) and 54.04 nm (PDI value 0.464, intercept 0.829), respectively (Fig. 3). This shows a difference in particle size between the two preparations, which might be related to pH changes.

**Figure 4:**
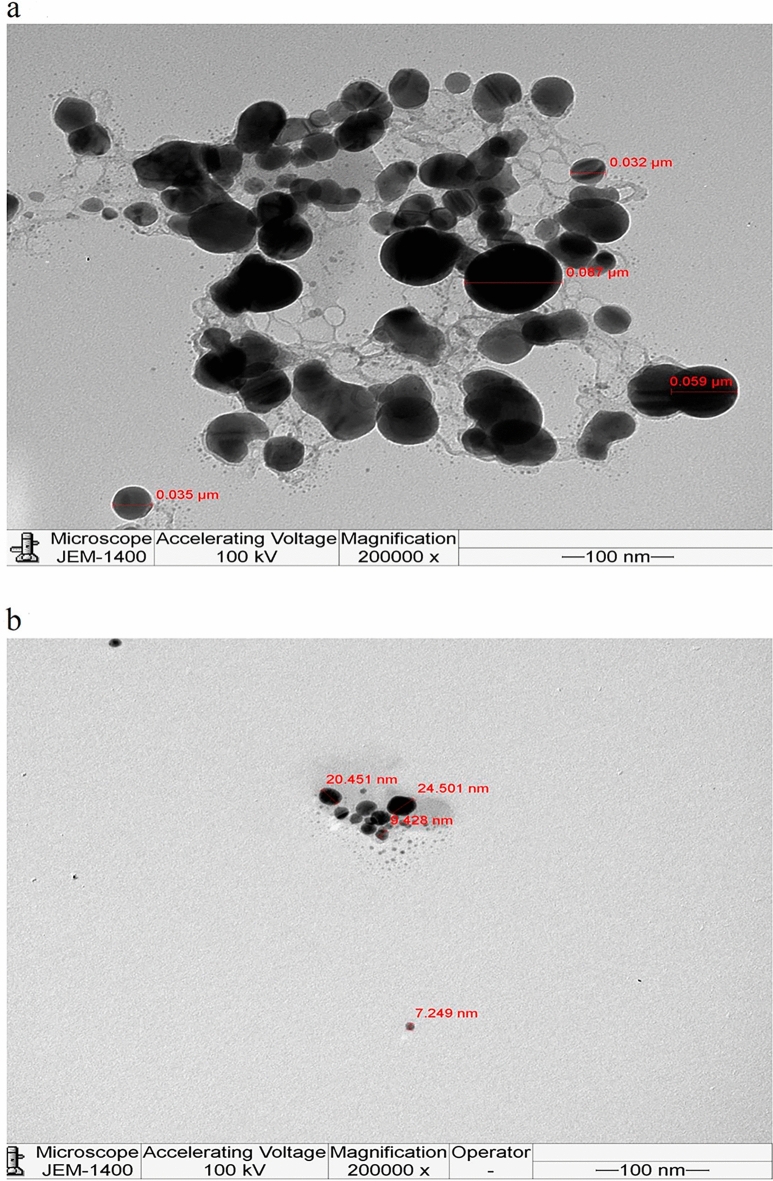
TEM microphotographs of *R. stricta* extract-biosynthesized AgNPs. JEM-1400 was used to quantify particle size at magnifications of 200,000. (**a**) Aq. AgNPs, (**b**) Alkaline Aq. AgNPs.

The two AgNPs preparations from *R. stricta* were analyzed by TEM imaging to corroborate the results of the zeta potential study. The findings revealed that both AgNPs were spherical in form, widely dispersed, and exhibited no aggregation (Fig. 4). The average diameter of Aq. AgNPs nanospheres ranged from 32 to 87 nm, whereas the average diameter of Alkaline Aq. AgNPs were 7.3–24.5 nm.

### *Fungistatic properties of R. stricta* aqueous preparations

The antifungal effects of *R. stricta* aqueous extract and biosynthesized AgNPs against plant diseases such as *D. halodes, D. tetramera, M. phaseolina, A. alternata,* and *C. australiensis* were tested on a PDA medium. The suppression of mycelial growth was measured in all treatments and compared to positive and negative controls by measuring the diameter of clear zones in PDA dishes.

**Figure 5:**
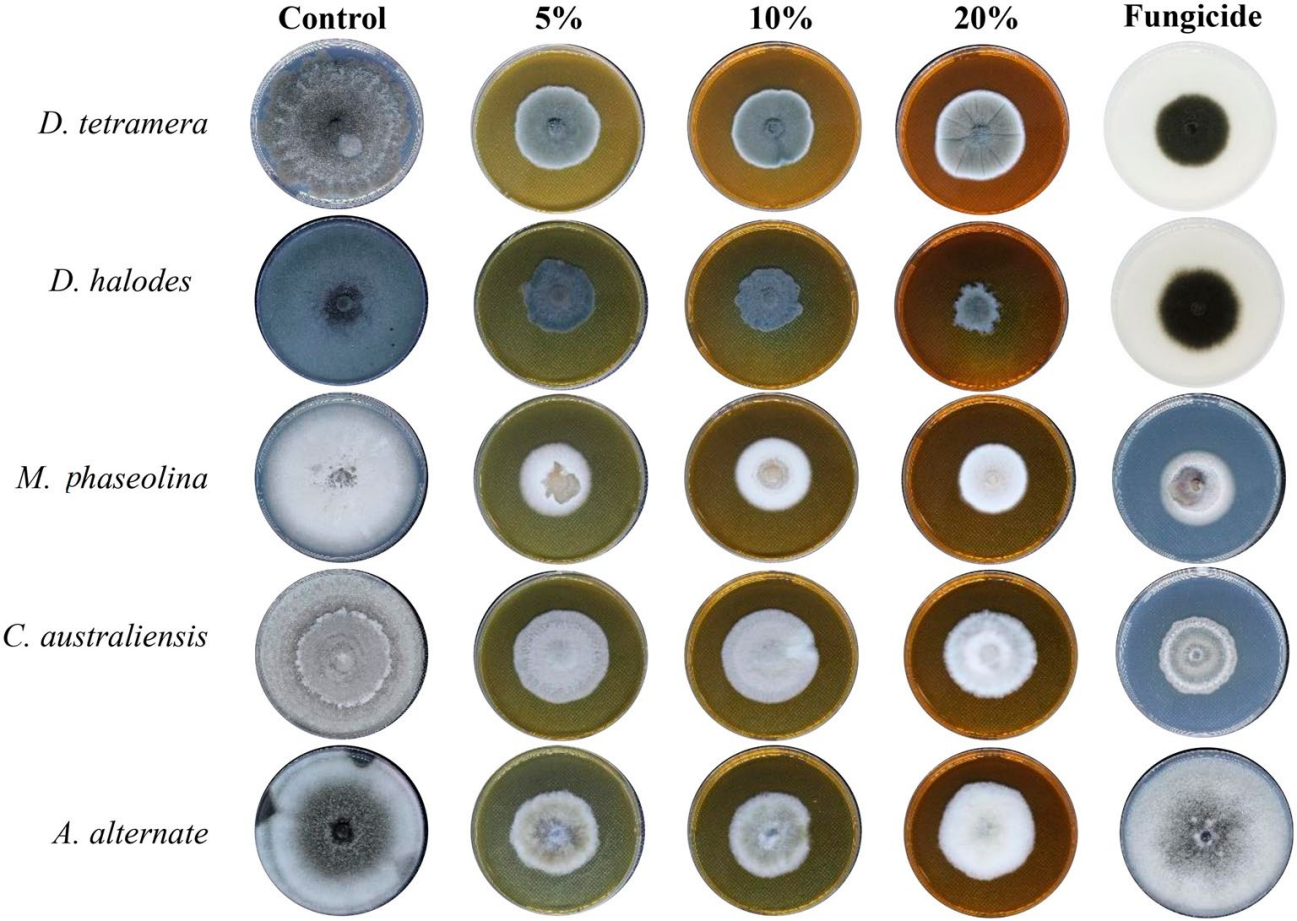
The antifungal activity of *R. stricta* Aqueous extract.

As shown in Fig. 5, the antifungal activity of the aqueous extract was at its maximum with a concentration of 20%. *D. halodes* was the most sensitive species to the fungicidal effects of all concentrations of *R. stricta* aqueous. On the other hand, *C. australienses* was the most resistant among all species (*P* < 0.001). Previcur energy had similar activities; however, it didn’t affect the mycelial growth of *A. alternate* (Fig. 5, Table 2).

**Table 2:** Assessment of In vitro antifungal activity of *R. stricta* aqueous extract.

Conc	0%	5%	10%	20%	Previcur energy
*D. tetramera*	66.4 ± 0.5	42 ± 0.1	29.8 ± 0.2	28.4 ± 0.7	31.2 ± 0.6
IMG%	0	36.7	55.1	57.1	53.1
*P-value*	–	< 0.01**	< 0.001***	< 0.001***	< 0.001***
*D. halodes*	80.0 ± 0.1	31.2 ± 0.2	24.4 ± 0.1	19 ± 0.8	32.5 ± 0.2
IMG%	0	61	69.5	76.3	59.3
*P-value*	–	< 0.001***	< 0.001***	< 0.001***	< 0.001***
*M. phaseolina*	70.5 ± 0.9	33.9 ± 0.1	32.5 ± 0.4	31.2 ± 0.6	32.5 ± 0.3
IMG%	0	51.9	53.8	55.8	53.8
*P-value*	–	< 0.001***	< 0.001***	< 0.001***	< 0.001***
*C. australiensis*	80.0 ± 0.2	44.7 ± 0.5	43.4 ± 0.8	39.3 ± 0.2	33.5 ± 0.5
IMG%	0	44.1	45.8	50.8	59.3
*P-value*	–	< 0.001***	< 0.001***	< 0.001***	< 0.001***
*A. alternata*	80.0 ± 0.5	39.3 ± 0.3	39.3 ± 0.2	25.8 ± 0.2	80.0 ± 0.2
IMG%	0	50.8	45.2	67.8	0
*P-value*	–	< 0.001***	< 0.001***	< 0.001***	–

*Significant.

**Figure 6:**
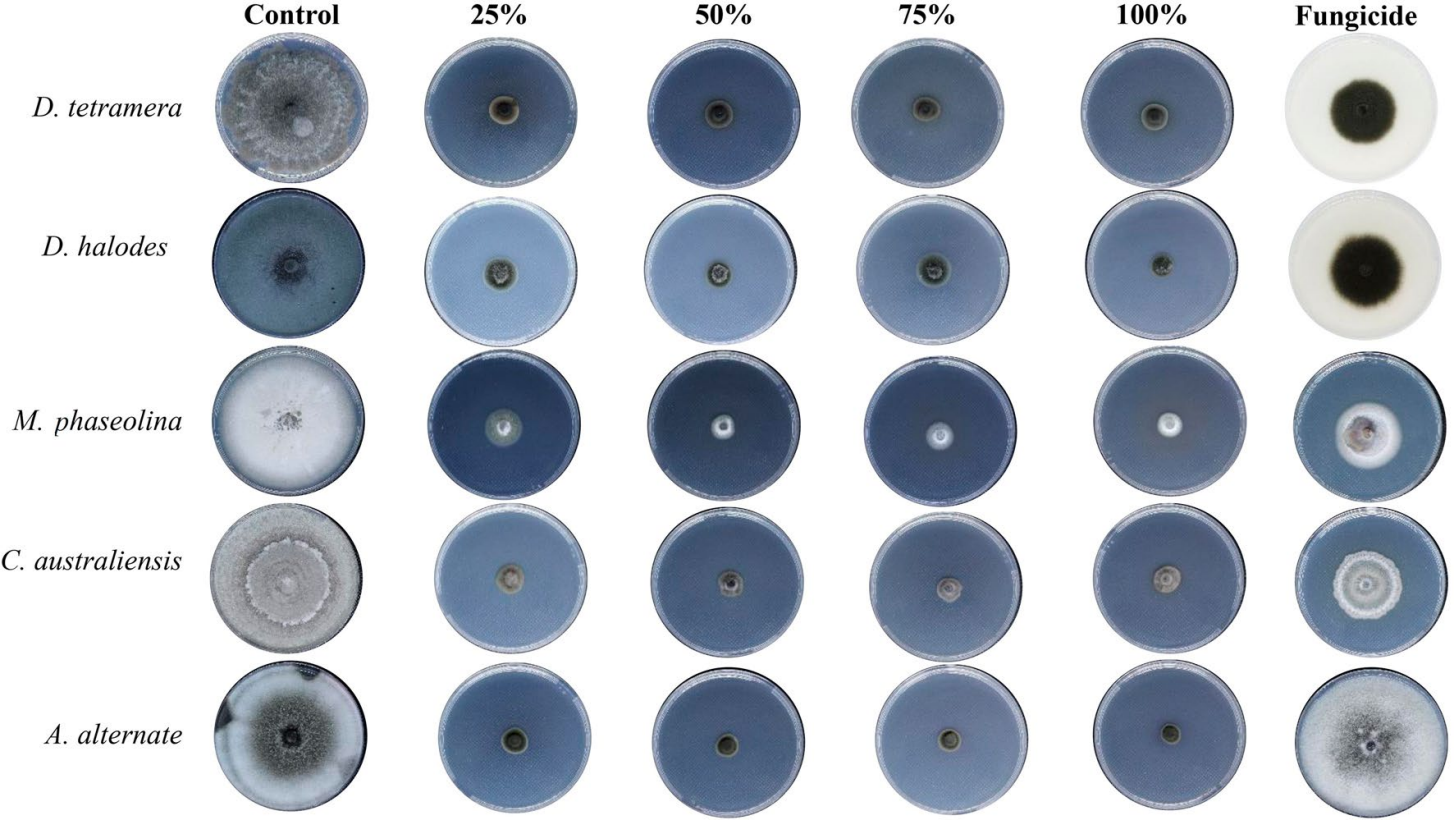
The antifungal activity of *R. stricta* Aq. AgNPs.

Treatment with Aq. AgNPs showed stronger antifungal activities against all species. As shown in Fig. 6, all concentrations almost stopped the mycelial growth of all species compared to the negative control. The calculated percentages of the mycelial growth inhibition (IMG%) of *A. alternata* had the maximum IMG% (100%) followed by *D. tetramera* (95.9%), *C. australiensis* (93.4%), *D. halodes* (91.5%), and *M. phaseolina* (90.4%) at the 100% dose of Aq. AgNPs (*P* < 0.001) (Table 3).

**Table 3:** Assessment of In vitro antifungal activity of *R. stricta* Aq. AgNPs.

Conc	0%	25%	50%	75%	100%	Previcur energy
*D. tetramera*	66.4 ± 0.5	8.1 ± 0.3	5.4 ± 0.2	4.1 ± 0.6	2.7 ± 0.1	31.2 ± 0.6
IMG%	0	87.5	91.8	93.9	95.9	53.1
*P-value*	–	< 0.001***	< 0.001***	< 0.001***	< 0.001***	< 0.001***
*D. halodes*	80.0 ± 0.1	9.5 ± 0.1	8.1 ± 0.3	7.5 ± 0.7	6.8 ± 0.7	32.5 ± 0.2
IMG%	0	88.1	89.8	90.7	91.5	59.3
*P-value*	–	< 0.001***	< 0.001***	< 0.001***	< 0.001***	< 0.001***
*M. phaseolina*	70.5 ± 0.9	13.6 ± 0.9	12.2 ± 0.3	8.1 ± 0.3	6.8 ± 0.9	32.5 ± 0.3
IMG%	0	80.8	82.7	88.5	90.4	53.8
*P-value*	–	< 0.001***	< 0.001***	< 0.001***	< 0.001***	< 0.001***
*C. australiensis*	80.0 ± 0.2	9.5 ± 0.2	6.1 ± 0.1	5.4 ± 0.2	5.3 ± 0.1	33.5 ± 0.5
IMG%	0	88.01	92.4	93.2	93.4	59.3
*P-value*	–	< 0.001***	< 0.001***	< 0.001***	< 0.001***	< 0.001***
*A. alternata*	80.0 ± 0.5	8.1 ± 0.5	5.4 ± 0.2	4.1 ± 0.6	0	80.0 ± 0.2
IMG%	0	89.8	93.2	95	100	0
*P-value*	–	< 0.001***	< 0.001***	< 0.001***	< 0.001***	–

*Significant.

**Figure 7:**
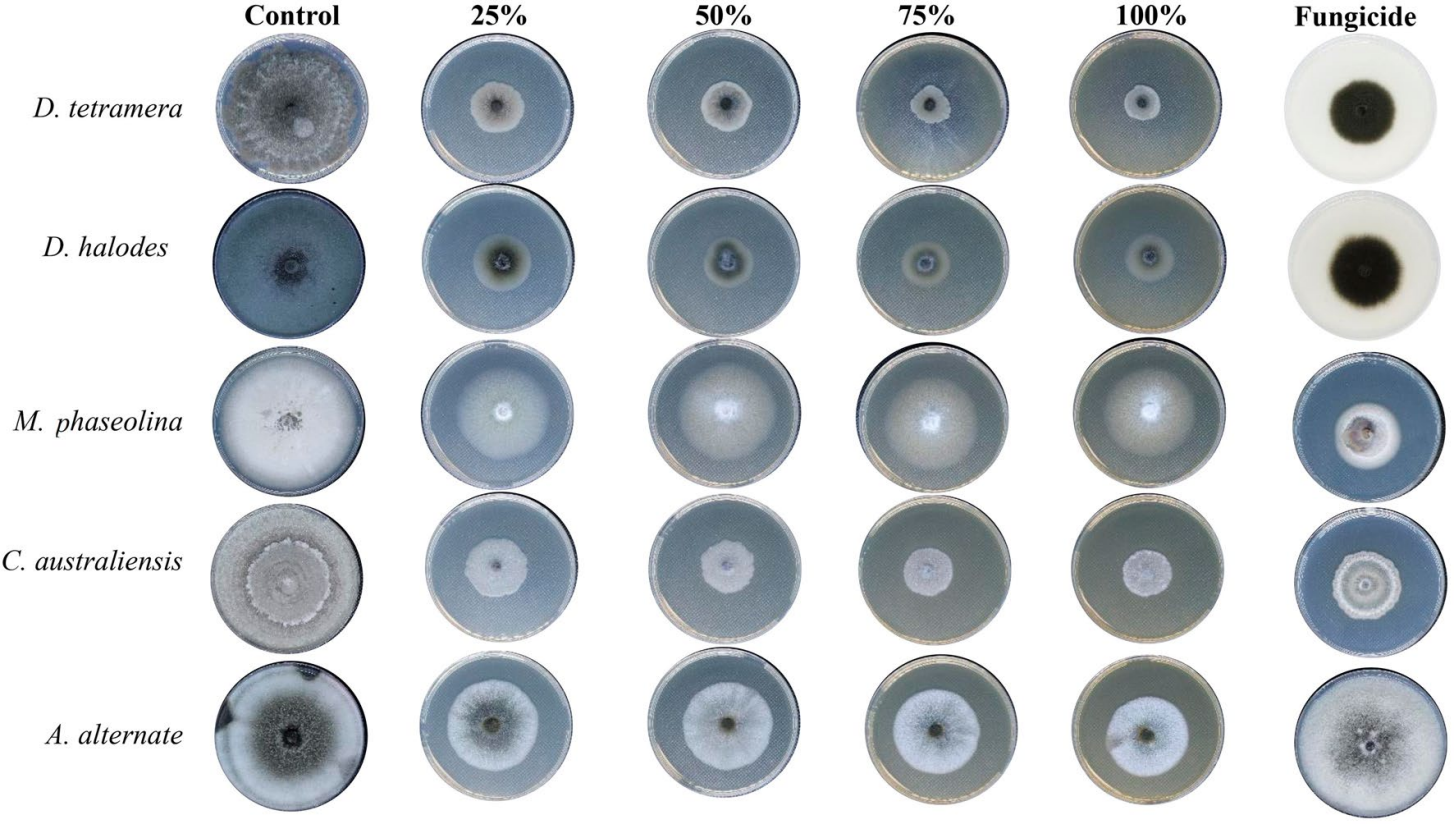
The antifungal activity of *R. stricta* Alkaline Aq. AgNPs.

**Figure 8:**
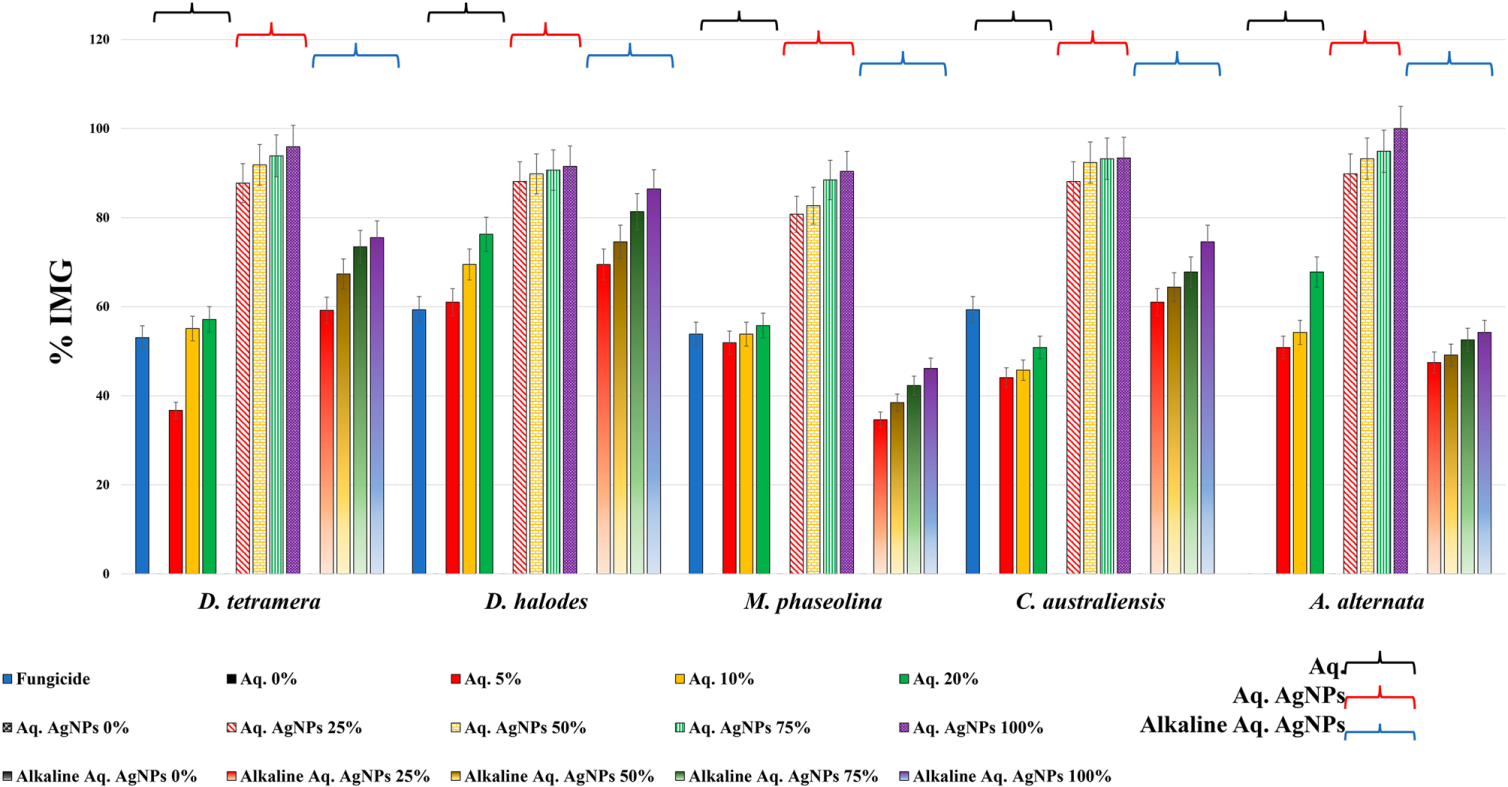
A summary of the antifungal effects induced by different treatments of *R. strica* extract on the mycelial growth of tested species.

Treatment with Aq. AgNPs showed stronger antifungal activities against all species. As shown in Fig. 7, various concentrations had variable effects on the mycelial growth of all species compared to the negative control. The calculated IMG% was the maximum for *D. halodes* (86.4%), followed by *D. tetramera* (75.5%), *C. australiensis* (74.8%), *A. alternata* (54.2%), and *M. phaseolina* (46.2%) at the 100% dose of Aq. AgNPs (*P* < 0.001) (Table 4). A summary of the different antifungal effects of tested preparations of *R. stricta* is shown in Fig. 8. It was revealed that Aq. AgNPs were the most effective fungicide among all treatments, while the crude extract had the weakest antifungal effects except for *M. phaseolina* and *A. alternata* compared to the Alkaline Aq. AgNPs. Among all of the tested species, it seems like *A. alternata* was the most sensitive to the highest concentrations of Aq. AgNPs, while *D. halodes* was the most sensitive species to the treatment with the crude aqueous extract and Alkaline Aq. AgNPs of *R. stricta.* Controversially, *M. phaseolina* was statistically the most resistant to the inhibitory effects of all treatments, compared to the negative control.

**Table 4:** Assessment of In vitro antifungal activity of *R. stricta* Alakine Aq. AgNPs.

Conc	0%	25%	50%	75%	100%	Previcur energy
*D. tetramera*	66.4 ± 0.5	27.1 ± 0.2	21.7 ± 0.5	17.6 ± 0.2	16.3 ± 0.1	31.2 ± 0.6
IMG%	0	59.2	67.3	73.5	75.5	53.1
*P-value*	–	< 0.001***	< 0.001***	< 0.001***	< 0.001***	< 0.001***
*D. halodes*	80.0 ± 0.1	24.4 ± 0.6	20.3 ± 0.3	14.9 ± 0.1	10.8 ± 0.4	32.5 ± 0.2
IMG%	0	69.5	74.6	81.4	86.4	59.3
*P-value*	–	< 0.001***	< 0.001***	< 0.001***	< 0.001***	< 0.001***
*M. phaseolina*	70.5 ± 0.9	46.1 ± 0.1	43.4 ± 0.9	40.7 ± 0.8	37 ± 0.1	32.5 ± 0.3
IMG%	0	34.6	38.5	42.3	46.2	53.8
*P-value*	–	< 0.01**	< 0.01**	< 0.001***	< 0.001***	< 0.001***
*C. australiensis*	80.0 ± 0.2	31.2 ± 0.6	28.5 ± 0.5	25.8 ± 0.2	20.3 ± 0.3	33.5 ± 0.5
IMG%	0	61	52.3	67.8	74.8	59.3
*P-value*	–	< 0.001***	< 0.001***	< 0.001***	< 0.001***	< 0.001***
*A. alternata*	80.0 ± 0.5	42 ± 0.3	40.7 ± 0.8	38 ± 0.1	36.6 ± 0.1	80.0 ± 0.2
IMG%	0	47.5	46.2	74.6	54.2	0
*P-value*	–	< 0.001***	< 0.001***	< 0.001***	< 0.001***	–

*Significant.

### Ultra-structural changes induced by AgNPs of* R. stricta*

**Figure 9:**
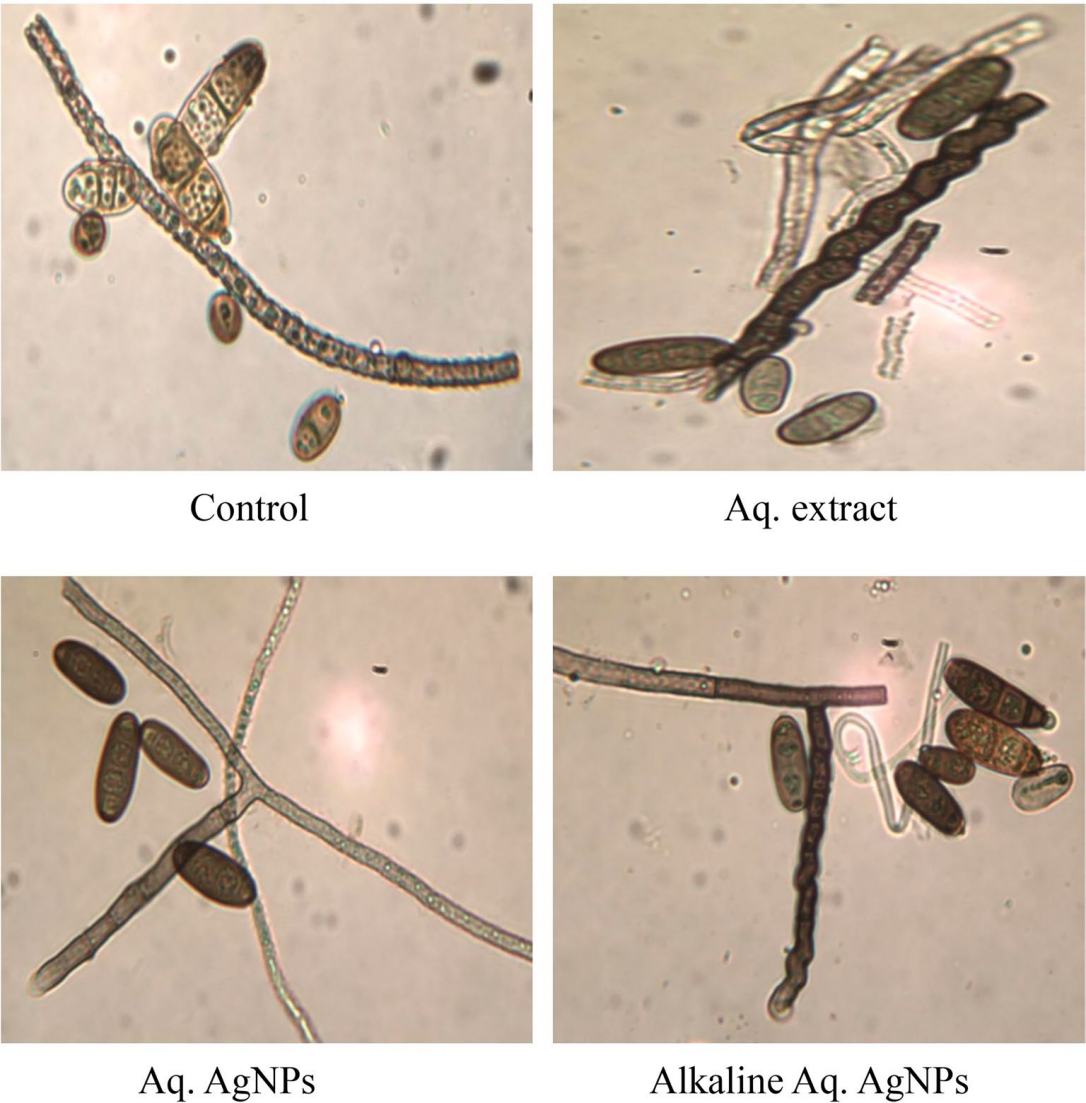
Light microscopic images displayed the morphological changes of *D. tetramera* in response to treatment with *R. stricta* aqueous extract and biosynthesized AgNPs at 40 × magnification.

Light microscopy imaging was used in the current investigation to compare the morphological changes in treated samples to the untreated control. All investigated fungal strains showed variances in the natural shape and size of hyphae and conidiophores. The morphological analysis of untreated *D. tetramera* showed light brown conidiophores, semi-elongated ellipsoid, cylindrical, and with round to oval ends. They are septate with no more than three pseudoseptates. The hyphae were brownish, granulated, unbranched, and with slight winding walls. Treatments with various preparations of *R. stricta* affected the septation and cylindrical shape of conidiophores, which appeared more oval, darker, and less viable than the control. The hyphae looked smoother, zigzag-shaped, swollen (in the case of crude aqueous extract), semi-branched (in the case of biosynthesized AgNPs), and bale-brown in Aq. AgNPs treatment (Fig. 9).

**Figure 10:**
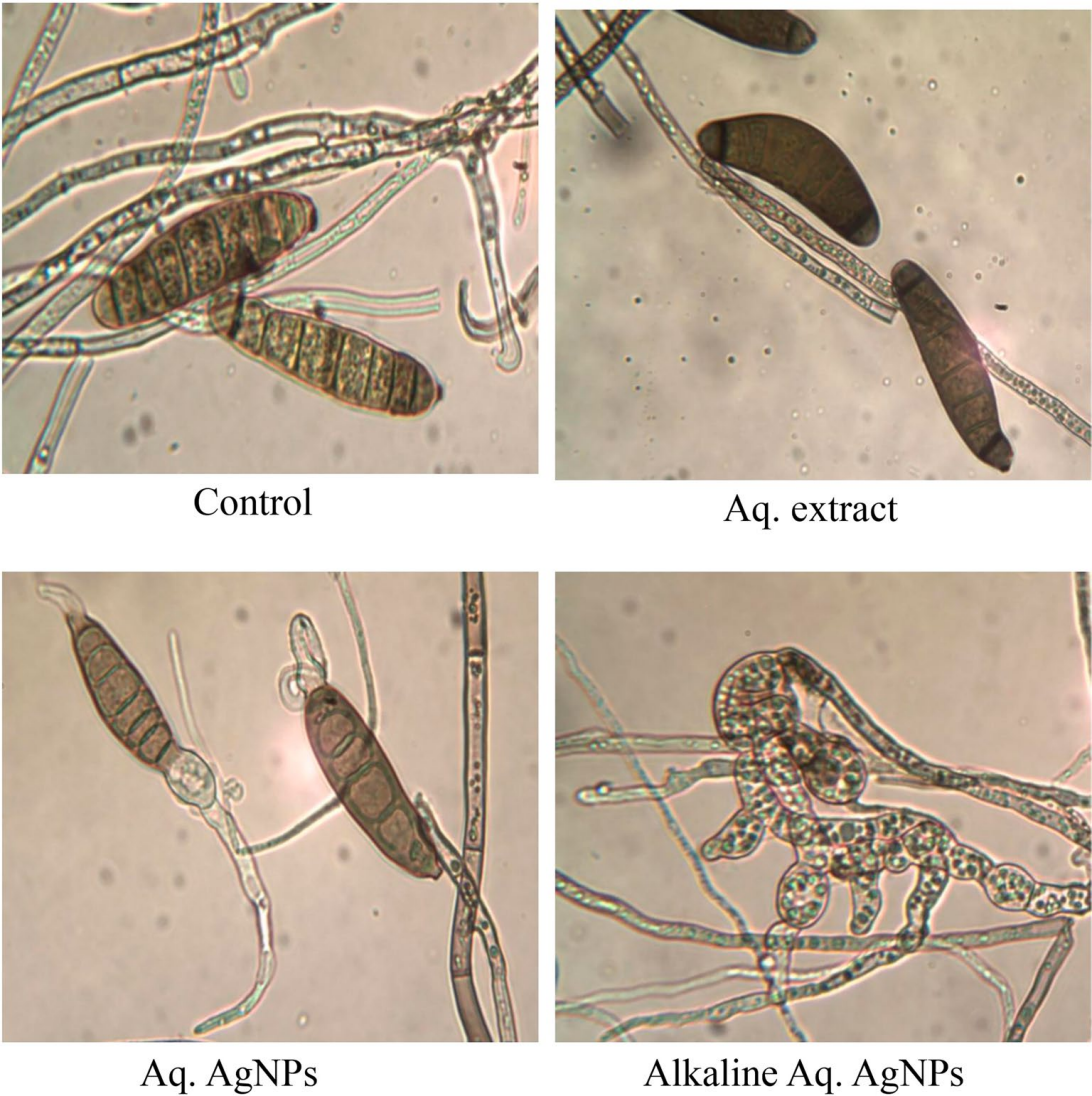
Light microscopic images displayed the morphological changes of *D. halodes* in response to treatment with *R. stricta* aqueous extract and biosynthesized AgNPs at 40 × magnification.

The morphological analysis of untreated *D. halodes* showed the light brown, subcylindrical conidia with smooth, elongated walls. The geniculated conidiophores are septate transversally and have distinct septa at the basal cells with 6–8 pseudosepta. The hilum appeared distinctly protuberant and unbranched. Images of different treatments of *R. stricta* showed the conidia darker and less septate than in the control. The hyphae looked smoother, vacuolated, swollen, and semi-branched (in the case of crude biosynthesized AgNPs), budding, thicker, and bale-brown (in the case of Alkaline Aq. AgNPs treatment) (Fig. 10).

**Figure 11:**
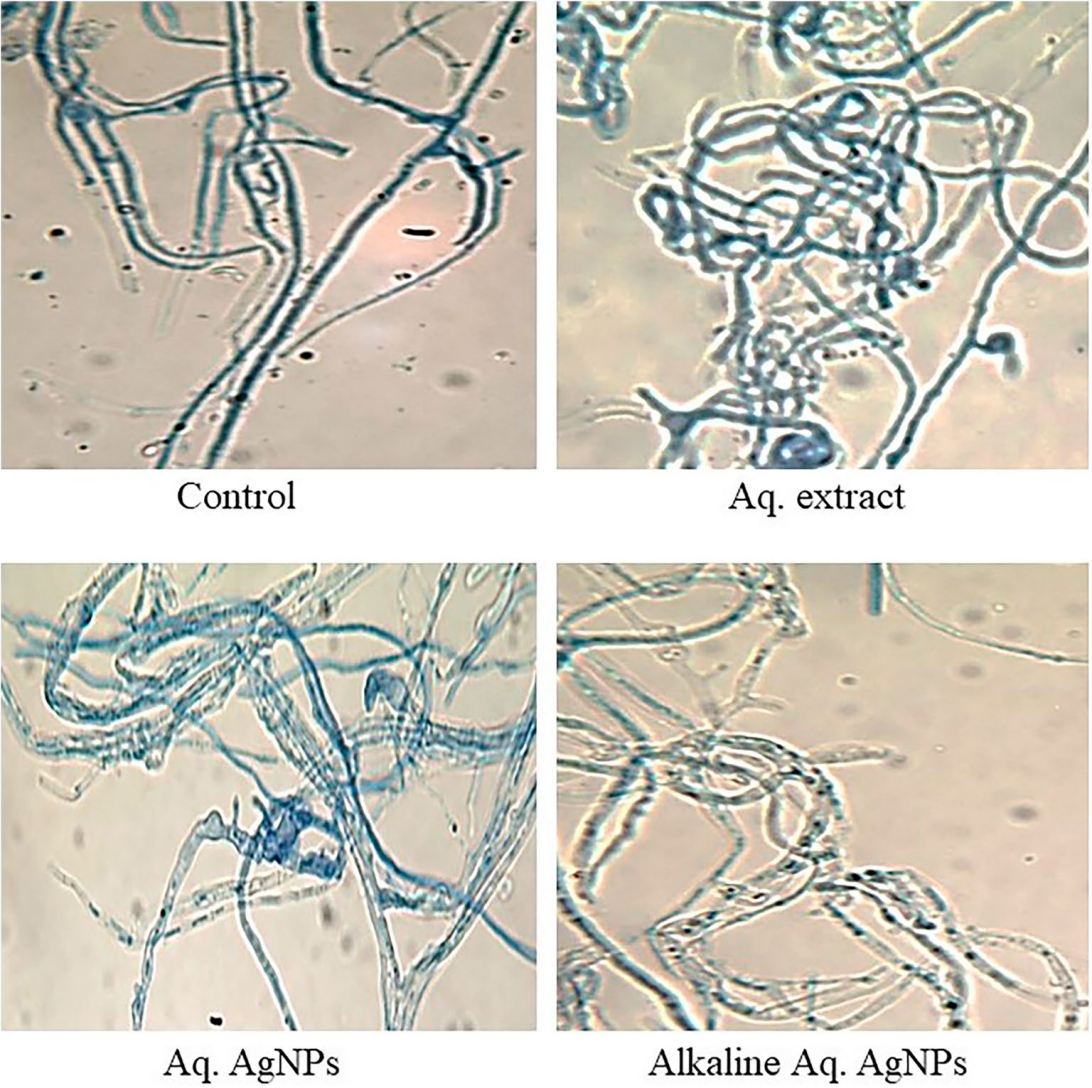
Light microscopic images displayed the morphological changes of *M. phaseolina* in response to treatment with *R. stricta* aqueous extract and biosynthesized AgNPs at 40 × magnification.

The light microscopic analysis of untreated *M. phaseolina* revealed a distinct morphology. The hyphae were hyaline, thin-walled, septate, and branched at the right angle. The microsclerotia were hyphae that were hardened with compact masses and oblong-shaped. The crude aqueous extract of *R. strica* didn’t induce any clear changes, whereas both of the biosynthesized AgNPs induced the thickening and rapture of hyphae, which appeared bale and hardened (Fig. 11).

**Figure 12:**
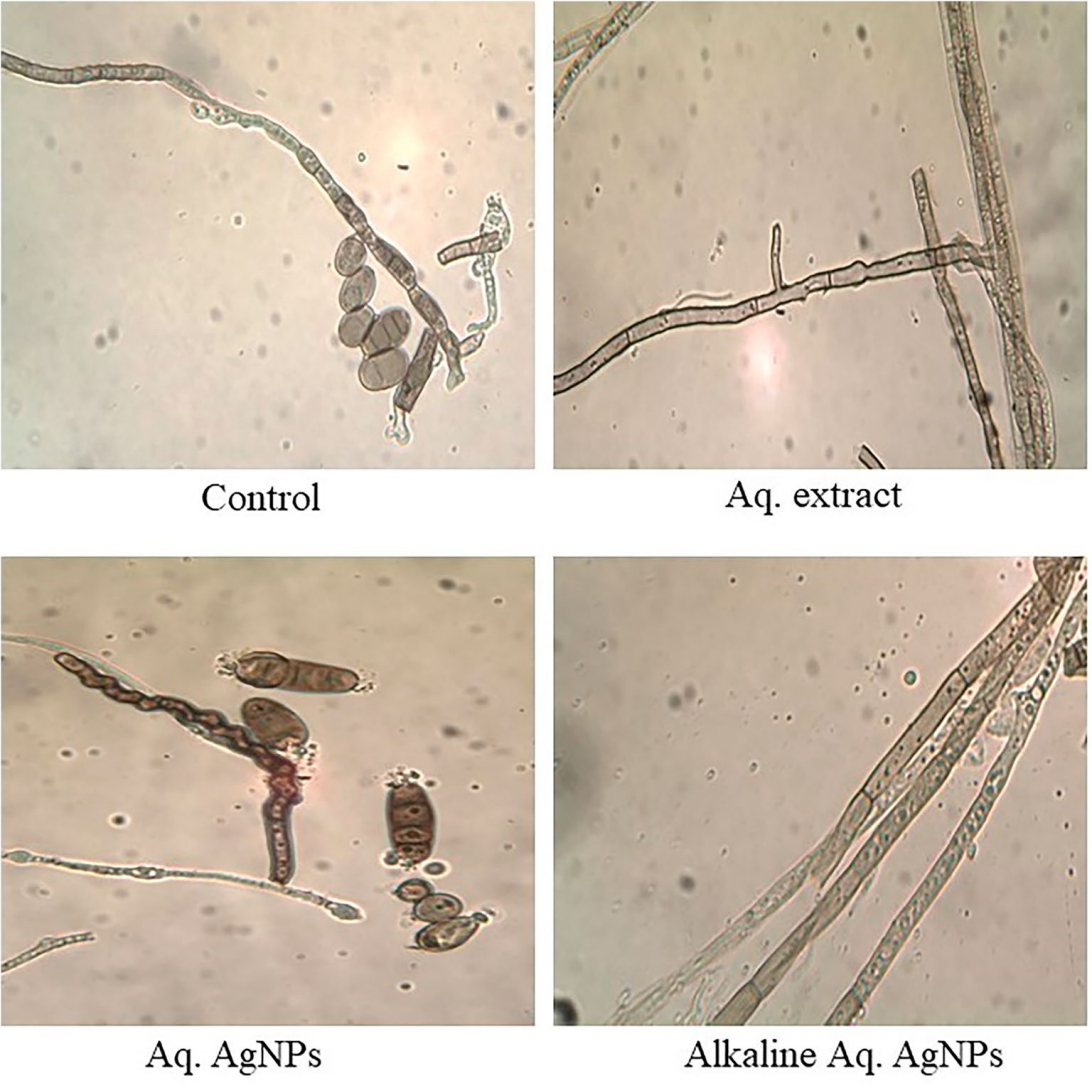
Light microscopic images displayed the morphological changes of *C. australiensis* in response to treatment with *R. stricta* aqueous extract and biosynthesized AgNPs at 40 × magnification.

The images of the untreated control of *C. australiensis* showed the sympodial, septate, flexuous, geniculated conidiophores with verrucose walls. The curved ellipsoidal conidia had rounded ends with four pseudosepta. The hyphae were protuberant, subhyaline, flexuous, light brown, and septate, with swollen and darker basal cells. The treatment with the crude aqueous extract of *R. stricta* or the Alkaline Aq. AgNPs didn’t reveal any significant changes in the hyphal structure, while the conidia didn’t appear. On the other hand, Aq. AgNPs induced extensive changes, where the hyphae were shorter, condensed, darker, and tighter than the control. The conidia appeared darker and raptured (Fig. 12).

**Figure 13:**
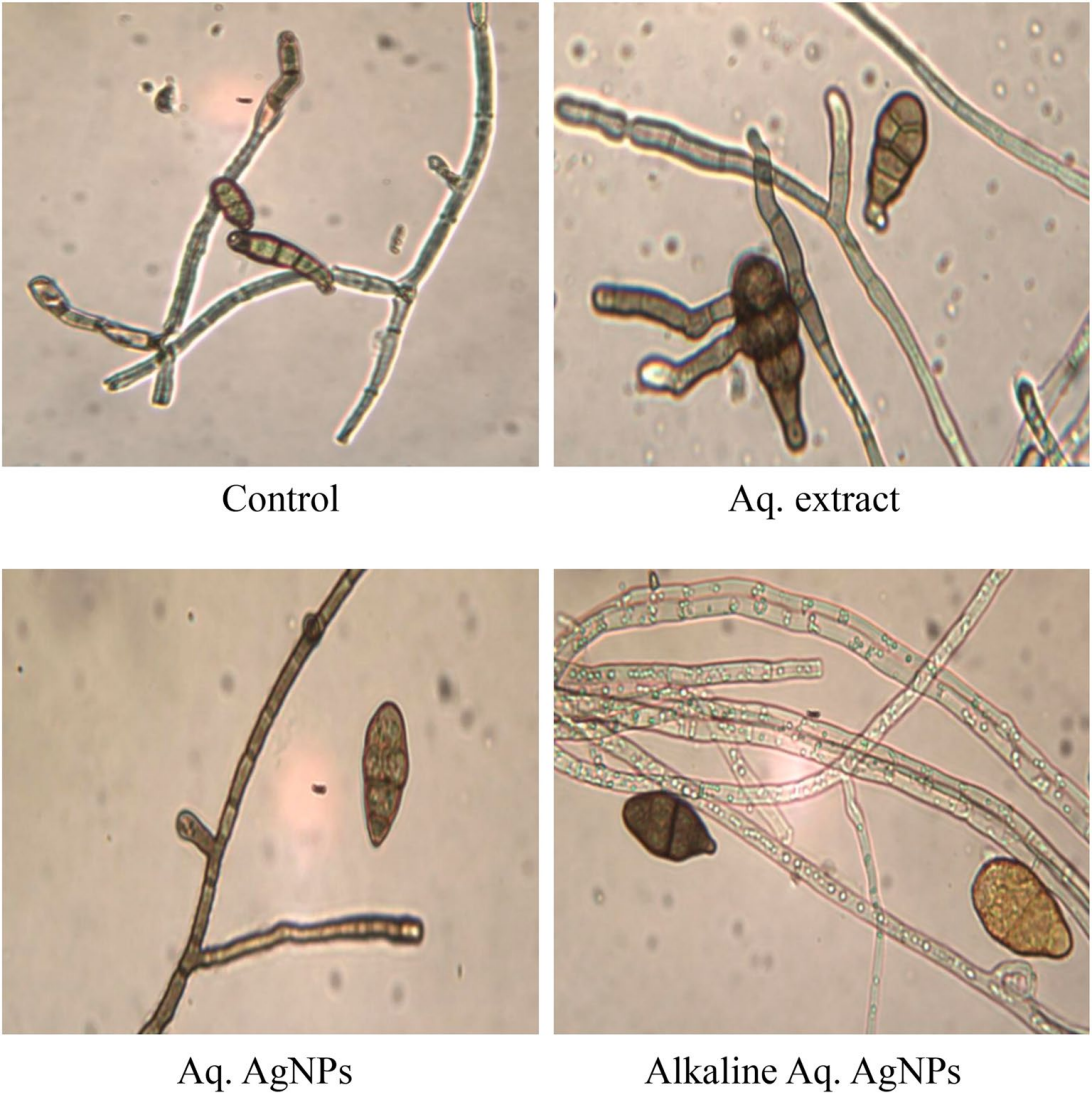
Light microscopic images displayed the morphological changes of *A. alternata* in response to treatment with *R. stricta* aqueous extract and biosynthesized AgNPs at 40 × magnification.

Finally, the microscopic analysis of *A. alternata* showed that the untreated fungus had clear morphology for both conidia and hyphae. The conidia were reddish-brown, ellipsoidal with a cylindrical beak, septate with 3–4 pseudosepta, and had verrucose smooth walls. The hyaline hyphae were multicell, septate, and branched. Different treatments of *R. stricta* caused the conidia to be more swollen, darker (in the case of crude extract and Aq. AgNPs), non-septate (in the case of Alkaline Aq. AgNPs), and oval to circular-shaped. The hyphae were more bale, transparent, and granulated in the species treated with the Alkaline Aq. AgNPs of *R. stricta* (Fig. 13).

**Figure 14:**
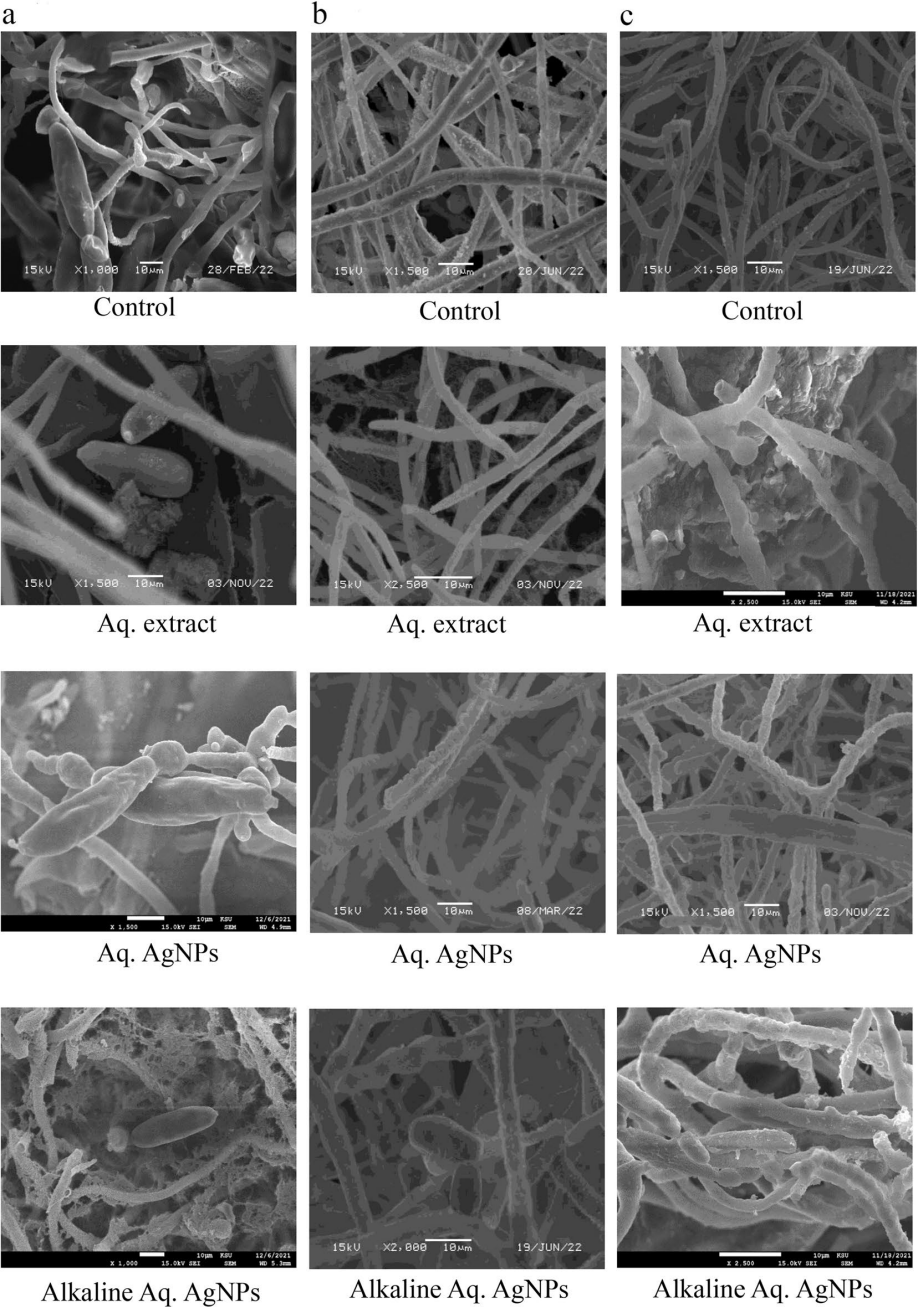
SEM microscopic images displayed the morphological changes of some of the tested fungi in response to treatment with *R. stricta* aqueous extract and biosynthesized AgNPs. (**a**) *D. halodes,* (**b**) *D. tetramera,* and (**c**) *C. australiensis.*

In the current study, we employed SEM to explore the morphological properties of the some of the susceptible species to the treatments under consideration. We show the species of *D. halodes, D. tetramera,* and *C. australiensis* here. The hyphae were deformed after treatment with Alkaline Aq. AgNPs, while the spores resembled the untreated control. After treatment with the aqueous extract and both biogenic AgNPs, there was an obvious contraction in hyphae and deformed spores of *D. halodes.* When compared to the natural structure exhibited in the untreated sample, these deformed spores had irregular forms and smaller hyphae (Fig. 14a). Without treatment, the *D. tetramera* control sample revealed the fungus's original structure. However, significant hyphae deformation was seen after treatment with the aqueous extract and both biogenic AgNPs. When treated with Aq. AgNPs, the fungal spores were deformed relative to the control and were essentially missing (Fig. 14b). *C. australiensis* control showed the fungus in its optimum form without treatment. Significant damage was detected after treatment with *R. stricta* aqueous extract and biosynthesized AgNPs. The hyphae appear to be cemented together, which might be explained by the fungus' capacity to make hydrophobic glue as a way of resistance to treatment. In comparison to the control, the detected spores look deformed and abundant (Fig. 14c).

## Discussion and conclusion

Nanotechnology is defined as the creation, display, manipulation, and use of nanoparticles^22^. Nanoparticles have a single dimension ranging from 1 to 100 nm. They have unique properties that set them apart from bulk materials, which might be affected their shape and size^23^. Solar cells, photovoltaic devices, heterogeneous catalysts, and pharmaceuticals have all benefited from their use^22,23^. Nanoparticles may exhibit improved or completely unique characteristics when specified qualities (such as size, shape, and structure) are considered. Because of their excellent properties and flexibility, gold and silver (noble metal) nanoparticles have piqued the interest of researchers^24^.

In the current study *R. stricta* aqueous extract and the alkaline fraction were used to produce various AgNPs. For measuring and comprehending the biological activity of biogenic AgNPs, comprehensive characterization was necessary. AgNPs size, form, size distribution, and aggregation are among these differentiating properties^25^. FTIR, UV–vis, Zeta potential, and TEM analysis were used to assess the properties of the biosynthesized AgNPs.

In the current study, the UV–visible spectrum of AgNPs was investigated, and large peaks were discovered to give surface Plasmon resonance (SPR) of AgNPs formed from aqueous extract and aqueous alkaline fraction of *R. stricta* leaves at 405 nm and 415 nm, respectively. The observed elevated shift of the UV peak might be ascribed to nanoparticle agglomeration generated by AgNPs assembly and the presence of various secondary metabolites in the reaction solution that interact with the silver nitrate^25^. Previous research has revealed that AgNPs biosynthesized from *R. stricta* aqueous extract had broad peaks at 405–420 nm^26–28^. In the study conducted by Rahman and colleagues (2023), the AgNPs produced from the aqueous extract of *R. stricta* whole plant had a specific peak at 420–450, which decreased to 305 nm after 30 min of UV exposure^29^. Another study indicated that the ZnO nanoparticles synthesized from the aqueous leaf extract of *R. stricta* had an SPR of 335 nm^30^.

FTIR is a very reliable analytical technique for detecting and displaying molecules' components, chemical structure, chemical bonds, functional groups, and bonding patterns^31^. FTIR analysis is a valuable method for studying the functional moieties involved in metal ion-biomolecule interactions^32^. In the current study, both of the biosynthesized AgNPs were analyzed using FTIR and compared to the crude aqueous extract to identify the compounds that function as stabilizing and coating agents, as well as to detect silver ion reduction. The FTIR spectra of various *R. stricta’s* aqueous leaf extract preparations indicated that they were high in hydroxyl groups, methylene, aromatic rings, methyl, and cyclic ether functional groups. Extra functional groups for nitrile, secondary alcohol, peroxide, conjugated ketone, and amines were added to the biosynthesized AgNPs. The changes in the plant extract's functional groups confirm that it serves as a reducing and capping agent in the production of AgNPs. In accordance with these findings, a previous study showed that the FTIR spectrum of *R. stricta*'s aqueous leaf extract contained hydroxyl, carboxyl, saturated aldehydes, amides, ketones, ethers, and alcohol functional groups^29^. Another study employed FTIR to analyze the AgNPs biosynthesized from the methanolic root extract of *R. stricta* and reported the existence of hydroxyl, amino, amide, and carboxyl groups^33^. All of these studies, in addition to the current findings, evaluated that biological molecule act as capping and reducing agents in the AgNPs synthesis.

Dynamic light scattering has been frequently used to characterize AgNPs that are generated utilizing chemical components. It calculates the size of the AgNPs colloidal solution, which scatters light and indicates their dispersion size in the 3–10 m range^23^. Furthermore, by calculating the velocity of biosynthesized AgNPs, zeta potential analysis may be used to evaluate their surface charge and stability. The velocity of nanoparticles is determined by their movement towards electrodes under the influence of an applied electric field^34^. In the current study, the Zeta potential of AgNPs synthesized by the aqueous extract of *R. stricta* detected their velocity at − 27.7 mV and − 37.9 mV for the Aq. AgNPs and Alkaline Aq. AgNPs, respectively, which indicated their stability. Similar study showed that a zeta potential value of -24.1 mv indicated the long-term stability. colloidal nature, and high dispersion of the AgNPs biosynthesized from *Urtica dioica* Linn. Leaves^35^. Another study showed that the AgNPs biosynthesized from different extracts of *Tabernaemontana ventricose,* member of *Apocynaceae,* had a velocity of -30.1 mV with a particle size of 70 nm^36^. These negative values of zeta potential analysis support the stability and dispersity of AgNPs as a result of the negative-negative repulsion^35^.

The surface shape and size of AgNPs biosynthesized from the aqueous and alkaline fractions of *R. stricta* leaves were determined using TEM. The results showed that the biosynthesized AgNPs were well-dispersed, with average diameters of 21–90 nm for Aq. AgNPs and 7.2–25.3 nm for Alkaline Aq. AgNPs, respectively. The TEM findings for the Aq. AgNPs were similar to the Zeta analysis results; however, the TEM results for the Alkaline Aq. AgNPs showed substantial discrepancies. This might be owing to the zeta analysis's overestimation of aggregates (based on Rayleigh's assumption) and peak width, or the elimination of nanoparticle aggregation in TEM^37^. Previous research found that AgNPs produced from methanolic and aqueous extracts of *R. stricta* root extract were spherical in form with average diameters of 20–35 nm^29,33^.

In the current work, *R. stricta* leaf extract and biosynthesized AgNPs inhibited the tested fungus species significantly. Growth observation of all examined fungi revealed a considerable shift in terms of growth density, color change, and perceived growth weakening. In that context, AgNPs biosynthesized from *R. stricta* root extract shown promising antibacterial action against *B. subtilis* and *E. coli*^33^. Another study found that *R. stricta* extract contains indole alkaloids and triterpene derivatives as main constituents that act as stabilizing agents during nanoparticle synthesis, increasing the antimicrobial activity of AgNPs against *Klebsiella pneumoniae, Salmonella typhi*, and *B. subtilis*^27^. *R. stricta* is a rich source of alkaloids with a wide range of structures and activities. Hajrah et al. (2020) reported that some of the alkaloids isolated from *R. stricta*, such as akuammidine, rhazimanine, stemmadenine, strictanol, and tetrahydrosecaminediol, showed potential antimicrobial activity against different human pathogens such as *P. aeruginosa, E. coli, S. aureus,* and *C. albicans*^38^*.* Also, ethanolic extract of *R. stricta* fruit shown outstanding antibacterial action *against S. aureus, E. coli, P. aeruginosa, B. subtilis, Streptococcus pyogenes*, and *S. typhi*^39^.

Ahmed et al. (2018) isolated 27 monoterpene indole alkaloids from *R. stricta*, which demonstrated significant antifungal activity against *C. albicans, Candida lusitaniae, Candida guilliermondii, C. glabrata, Candida krusei*, and *Candida parapsilosis* with MIC values ranging from 3.125 to 50 g/m^40^. Furthermore, a previous study reported significant anti-fungal activities of different extracts against *Trichophyton longifusis, Aspergillus flavus, C. albicans,* and *Fusarium solani*^41^. For our knowledge, different extracts of *R. stricta* or the biosynthesized nanoparticles were examined for their antifungal activities against the tested species.

As a conclusion, our findings revealed that tested *R. stricta* extracts and fractions have antifungal efficacy against some plant pathogenic fungi. When compared to Alkaline Aq. AgNPs, the pre-synthesized Aq. AgNPs demonstrated high activity. That might be because the Alkaline aqueous AgNPs missed some of the functional groups which weakened its activity; however, of its significant fungicidal activity as compared to the untreated species. More experimental study on employing plant extracts as an alternative to toxic chemicals is required, as well as a deeper analysis of the ultra-cellular and molecular damage induced by *R. stricta* extract to elucidate its probable antifungal processes. Also, further research to explore the mechanisms underlying the antifungal activity of *R. stricta* different extracts and biosynthesized nanoparticles.

## Materials and methods

### Plant collection

Fresh leaves of *R. stricta* were obtained from the desert surrounding Riyadh, Saudi Arabia (latitude: 24°56′52" N, longitude: 45°42′37" E). The plant was identified at the Botany and Microbiology Department of the College of Science at King Saud University in Riyadh, Saudi Arabia, according to the Morphology Characteristics^42^. Experimental research on plant, including the collection of plant material, comply with the international guidelines and the Nagoya Protocol of the Convention on Biological Diversity (CBD), available from: https://www.cbd.int/doc/legal/cbd-en.pdf.

### Fungi species

In the current investigation, five fungus species were examined. The species were obtained from the American Type Culture Collection (ATCC) in Manassas, Virginia, USA. *D. halodes* (ATCC, 34172), *D. tetramera* (ATCC, 18957), *M. phaseolina* (ATCC, 64333), and *A. alternata* (ATCC, 66981) were among those identified. As previously reported^43^, *C. australiensis* was acquired and identified by the Botany and Microbiology Department, College of Science, King Saud University, Riyadh, Saudi Arabia. These strains were all cultivated in petri plates using 5.7% potato dextrose agar (PDA). The strains were either stored at 4 °C or sub-cultured once a month until they were used.

### Preparation of plant extracts

The leaves aqueous extract was made in the same manner as previously described^44^. Leaves were cleansed with distilled water, then shade-dried before being ground into a fine powder. 10 g were steeped in 100 ml distilled water and heated until ebullition occurred. Before filtering using Whatman filter paper No. 1, the three aqueous extracts were combined and filtered through muslin cloth. The filtrate was vacuum distilled at reduced pressure at 50 °C using a Rotavapor^®^ R-300 (BÜCHI Labortechnik AG, Flawil, Switzerland) to remove water. The filtrate was concentrated and lyophilized, yielding an aqueous extract with approximately 40 g of dark brown residue.

The alkaline fraction of *R. stricta* was created in the same way as it was reported earlier^45^. The residue of *R. stricta* filtration using Whatman filter paper was removed and filtered with 100 cc of 0.15 M NaOH at 80 °C for 30 min. To obtain the alkaline residue extract, the filtrate was neutralized with HCl, condensed, and lyophilized.

### Preparation of AgNPs

AgNPs were prepared as previously described by Shaik and colleagues (2018), with minor adjustments^46^. In brief, the *R. stricta* aqueous extract or alkaline fraction was combined, separately, in a 1:9 ratio with silver nitrate AgNO_3_ (1 mM). The mixture was gently boiled at 90°C with a magnetic stirrer (VELP scientica Srl, Usmate, Italy). During that phase, a dropper was used to add roughly 5 ml of 1N sodium hydroxide (NaOH) to the mixture, and the yellow hue of the liquid turned dark brown. The reaction was halted at that moment, and after cooling, the mixture was spun at 9000 rpm for 30 min, yielding a black precipitate. The filtrate was gently withdrawn, and the black residues were separated for manufacture of aqueous extract AgNPs (Aq. AgNPs) or Alkaline Aq. AgNPs. The precipitates were washed twice with deionized water, dried for 12 h at 80 °C, and kept at 4 °C for future use.

### Characterization of AgNPs

#### UV–visible (UV–Vis) spectroscopy

A Shimadzu UV–visible spectrophotometer (Tokyo, Japan) was used. The reduction of pure Ag + ions was measured at UV-245 double-beam (200–800 nm) according to the manufacturer's recommendations^44,47^.

#### Fourier-transform infrared spectroscopy (FTIR)

The infrared absorption and emission spectra of the macromolecules detected in the prepared test samples were measured using FT-IR spectroscopy^19^. The FTIR spectra of each produced nanoparticle were measured using a Nicolet 6700 FTIR spectrometer (Thermo Scientific, Waltham, MA, USA) with an absorption range of 400–4000/cm. The functional groups contained in each nanoparticle were identified via FTIR analysis. The spectrometer (Nicolet 6700) has a beam splitter and a detector (DTGS) with OMNIC software that was used to gather and analyze the spectra in the 400 to 4000/cm scan. As specified in the manufacturer's instructions, the IR spectra obtained were utilized to interpret the functional moieties contained in each biogenic AgNPs^22^.

#### Zeta potential analysis

The average hydrodynamic diameter of the biogenic AgNPs was determined using Dynamic Light Scattering (DLS) analysis in this work. In this case, the Zeta sizer instrument (Malvern Instruments Ltd., zs90, Worcestershire, UK) was used to determine the zeta potential value and hydrodynamic diameter of AgNPs, as directed by the manufacturer^22,48^.

#### Transmission electron microscopy (TEM)

A Transmission Electron Microscope (model JEOL JEM-1011, Peabody, MA, USA) was used to investigate the form and particle size distribution of the biosynthesized AgNPs. Each test sample was put in an 8-L container on a 300-mesh carbon-coated copper grid. Images were captured at an acceleration voltage of 200 kV^19,48^.

### Assessment of the antifungal activity

The poison plate method was used to assess the antifungal properties of *R. stricta* and biosynthesized AgNPs^49^. Four concentrations of crude aqueous extract (0, 5, 10, and 20% (w/v)) were made, whereas five concentrations of biosynthesized AgNPS (0, 25, 50, 75, and 100% (w/v)) were prepared. At a concentration of 53%, Previcur Energy (SL-840, Bayer Crop Science Ltd., Dublin, Ireland), a multi-site, broad-spectrum fungicide (Propamocarb) formulated to protect plants from many infectious diseases^50^, was employed as a positive control. 1 ml of each treatment was made in distilled water and filtered using a 0.45 m bacterial filter (MF-Millipore, Sigma-Aldrich, St. Louis, MO, USA). Then, 1 ml of each filtrate was gently mixed with 19 ml of the molten PDA and placed into a 60 cm^2^ Petri plate. For each concentration and species, a single plate was created. After solidification, a mycelial plug (6mm) of each of the tested fungus, none-days old, was centered in each plate in a sterile laminar flow cabinet. The cultures were incubated at 25 °C in dark. All tests were carried out in triplicate. The suppression of mycelial growth was estimated as follows:

$$ {\text{Mycelial Growth Inhibition }}\left( \% \right) \, = \, \left( {{\text{C}} - {\text{T}}} \right)/{\text{C }} \times {1}00, $$

where C represents the colony diameter in the control plate and T represents the colony diameter in the treated petri plates.

### Detection of the ultrastructural alterations in the treated species

The morphological and ultrastructure characterization of selected fungal growth in response to treatment with aqueous *R. stricta* extract and biosynthesized AgNPs was investigated.

#### Light microscopy

According to the manufacturer's instructions, the ECLIPSE Ni-E light microscope augmented with an F-mount camera with a digital sight of 50M (NIKON Corp., Tokyo, Japan) was employed here. A sterile spatula was used to take a disc of 6mm diameter from each mycelial growth and insert it in the center of a sterile slide. Lactophenol blue solution (Sigma-Aldrich, St. Louis, MO, USA) was used to stain the slides, which were then covered with sterile coverslips and inspected at 40 × power^51^.

#### Scanning electron microscope (SEM)

Some of the tested species were SEM scanned to evaluate the ultra-morphological alterations caused by different *R. stricta* treatments. The samples were examined using a JEOL JSM-6060LV scanning electron microscope equipped with a pre-centered W hairpin filament electron source (JEOL LTD., Tokyo, Japan). As previously stated, the slides were fixed overnight at 4°C with 2.5% Glutaraldehyde, then washed with phosphate buffer (pH 7.2), and re-fixed with 1% Osmium Tetroxide. As previously stated, dehydration in successive dilutions of ethanol, freeze-drying in a critical point drier, and mounting on gold-plated stubs were all completed^52^.

### Statistical analysis

For statistical purposes, the experimental experiments were carried out in triplicates. The statistical analysis was carried out using IBM's Statistical Package for the Social Sciences (SPSS) version 22 (Armonk, NY, USA). If the *P-Values* were less than 0.05, the results were considered significant.

## Data Availability

The raw data presented in this study are available from the corresponding author upon reasonable request.
